# G9a Is SETting the Stage for Colorectal Oncogenesis

**DOI:** 10.3390/genes11060616

**Published:** 2020-06-04

**Authors:** Christopher J. Bergin, Yannick D. Benoit

**Affiliations:** Department of Cellular and Molecular Medicine, University of Ottawa, Ottawa, ON K1H 8M5, Canada; cberg040@uottawa.ca

**Keywords:** colorectal cancer, epigenetics, G9a, driving mutations, cancer stem cells

## Abstract

Recently, Kato et al. reported recurrent activating mutations in the SET domain of histone methyltransferase G9a, driving an oncogenic cascade in melanoma. The authors also reported correlations between G9a expression and the regulation of the canonical WNT pathway. Although we could not observe such mutations in human colorectal adenocarcinoma, newly reported findings are of high relevance to colorectal cancer, as WNT target gene signatures were closely associated with G9a expression. Here, we put into perspective such new results on G9a expression in colorectal cancers and the potential relationship with tumor heterogeneity and acquisition of neoplastic stemness.

Colorectal cancer is a heterogeneous disease associated with accumulation of various genetic and epigenetic aberrations. The epigenetic landscape in colorectal tumors is key to establishing an intratumoral cellular hierarchy that dictates tumorigenesis [1]. Thus, chromatin regulators are now acknowledged as key drivers of oncogenesis, either through direct genetic alterations and/or changes in their expression or catalytic activity [2]. The case of the histone methyltransferase EZH2 is particularly interesting, as it was reported to be overexpressed in colorectal tumors vs. normal intestine, and associated with tumor-initiating functions [3]. Loss of H3K27 methylation by inactivation of polycomb repressive complex-2 was shown to induce apoptosis in colon tumor-initiating cells several years ago [4]. However, direct inhibitors of EZH2 were only confirmed recently as a powerful tool to suppress such a function in vivo [3]. Lately, another SET-domain histone methyltransferase has attracted significant attention regarding colorectal carcinogenesis. G9a is a histone methyltransferase forming a heterodimeric complex with G9a-like protein (GLP), responsible for the mono and di-methylation of H3K9 and generally associated with transcriptional repression. Luo et al. previously reported that G9a expression was required to preserve the in vivo tumor initiation capacity of human colorectal cancer cell lines [5]. Moreover, observations made from patient tissues linked G9a expression with the acquisition of resistance to radiation therapy in colorectal tumors [5].

In a recent issue of *Cancer Discovery*, Kato et al. identified recurrent activating mutations and copy gains in *EHMT2*, which encodes G9a, and such alterations were shown to drive oncogenesis in human melanoma [6]. Moreover, the authors associated G9a hyperactivity and overexpression with positive regulation of canonical WNT target genes in several cancers, including colorectal. Interestingly, a role for G9a in H3K9me2-mediated transcriptional repression of the soluble WNT inhibitor Dickkopf-1 (DKK1) was demonstrated. This supports observed correlations between WNT/β-catenin target gene signatures and pro-oncogenic G9a overexpression cancers, including colorectal tumors.

Similar to the authors, we used the cBioPortal platform to run EHMT2/G9a mutation and copy number analysis on TCGA PanCancer Atlas colorectal adenocarcinoma cohort (524 samples) [7]. We could not identify the recurrent gain-of-function mutations in the G9a SET domain (G1069L and G1069W) reported by Kato et al. in this set of patients (Figure 1A), as well as in Metastatic Colorectal Cancer samples (MSKCC, Cancer Cell 2018). EHMT2 gains/amplifications (≥3 copies) are found in 25.8% of melanomas and 21.1% of colorectal patients (Figure 1B, TCGA PanCancer Atlas Colorectal Adenocarcinoma). EHMT2 was found mutated in 1.7% of queried colorectal cancer patients (Figure 1C).

We expanded our analysis to other malignancies using TCGA PanCancer Atlas cohorts and assessed the EHMT2/G9a mutation status in breast (1084 samples), glioblastoma (592 samples), pancreatic (184 samples) and lung tumors (1053 samples). We could not identify recurrent G1069 mutations in the G9a SET domain as reported by Kato et al. Thus, to the best of our knowledge, reported activating mutations in G9a seem to be a distinct characteristic of melanomas. Yet, overexpression of G9a protein and/or associated H3K9 methylation levels is not anecdotic in colorectal tumors and has been reported by several groups [8,9,10].

Despite clear connections between G9a activating mutations, Kato et al. established a correlation between this histone methyltransferase and repression of DKK1 in colorectal tumors. This complements the body of knowledge on canonical WNT hyperactivity as a hallmark of colorectal cancer linked to tumorigenesis, self-renewal and maintenance of neoplastic stem phenotype. The interplay between G9a and soluble WNT inhibitors was previously documented in neuroendocrine tumors, where DKK1 and DKK3 promoters are silenced by G9a-dependent H3K9 methylation [11,12]. In addition, Kato et al. used ChIP-seq data from primary colon cancer-initiating cells to confirm G9a enrichment at the DKK1 promoter. This further supports the prospect of G9a as a modulator of canonical WNT/β-catenin in neoplastic stem cells. However, oncogenic mutations within the β-catenin destruction complex act downstream of the G9a-DKK1 axis in hyperactivation of the canonical WNT pathway [11]. While APC gene mutations are found in a large proportion of colorectal tumors and are tightly related to WNT hyperactivity, no particular patterns were observed between APC-mutated cases and G9a mutations or expression (Figure 1C,D). As stated by Kato et al., G9a could drive tumorigenesis by impacting WNT-related gene programs via other pathways, which is likely to be the case in colorectal cancer. Given the importance of G9a in early embryonic gene regulation [13,14,15], it could possibly influence pluripotent-like gene networks linked to cancer stemness [16].

Remarkably, Kato et al. addressed the issue of a significant number of patients experiencing resistance to immunotherapeutic approaches. Indeed, the use of immune checkpoint inhibitors has currently yielded no major clinical benefits for most colorectal cancer patients [17]. The authors described the inverse correlation observed between G9a expression/copy number and T-cell signatures in melanoma patients. Furthermore, the GLP/G9a inhibitor UNC0642 was used in vivo and led to substantial tumor regression when combined with anti-PD1 and anti-CTLA4 treatments in a syngeneic melanoma mouse model. The authors concluded that G9a overexpression promotes a poorly immunogenic tumor microenvironment through DKK1 and WNT signaling. Such a “cold” tumor immune microenvironment could be reversed by G9a inhibition. Tumor immune insusceptibility is another hallmark of cancer stemness, as recently reported by Miranda et al., who described the role of neoplastic stem cells on immune cell exclusion from the tumor microenvironment [18]. Thus, cancer stem cells contributing to intratumoral heterogeneity, are limiting tumor immune responses via silencing of endogenous retroviral elements and up-regulation of immunosuppressive checkpoints [18]. It is noteworthy that G9a is involved in epigenetic silencing of such endogenous retroviral elements in pluripotent stem cells [19]. Taken together, these findings suggest that G9a overexpression, as observed in colorectal cancer, may contribute to poor tumor immunogenicity by inducing a neoplastic stemness phenotype.

The findings reported by Kato et al. are setting the stage for the use of the G9a/GLP inhibitors as an opportunity to evaluate G9a inhibition as a therapeutic avenue to target colorectal cancer-initiating cells in vivo. As aforementioned, the small molecule UNC0642 was used to block G9a histone methyltransferase activity in mouse syngeneic and human-to-mouse xenograft models. The authors highlighted the importance of developing novel and more effective therapeutics targeting epigenetic determinants, emphasizing the particular “druggability” of G9a in cancer. Recently, Casciello et al. also used UNC0642 to show that inhibition of G9a in breast cancer cells decreases tumor growth and lung metastasis in vivo [20]. These are encouraging demonstrations of in vivo potential of UNC0642 to inhibit H3K9 methylation, since it was previously considered to have poor pharmacokinetics [21]. It will be interesting to see if more studies continue to implement a direct-G9a/GLP inhibition strategy in upcoming cancer research.

## Figures and Tables

**Figure 1:**
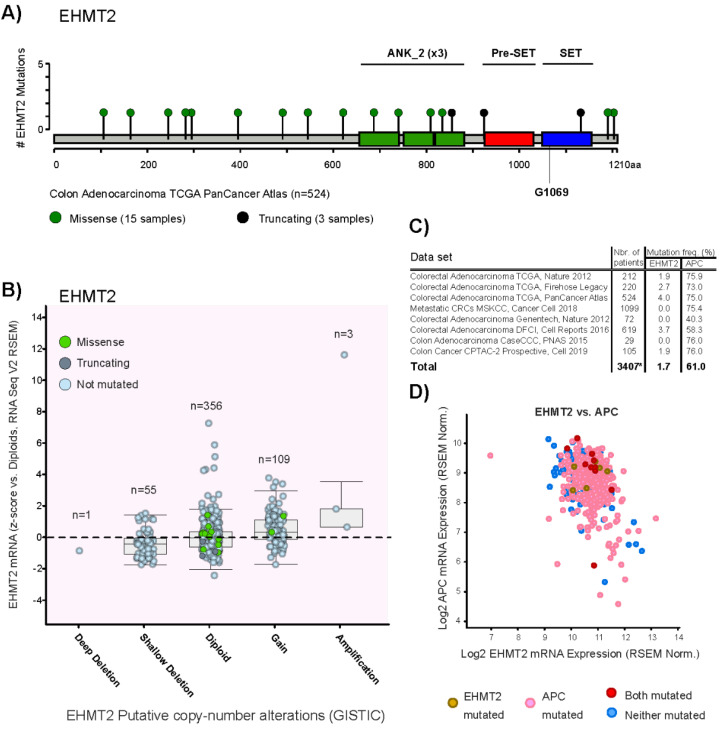
Mutational status of EHMT2/G9a and its relationship with mRNA expression in human colorectal tumors. (**A**) Domain architecture of the EHMT2 gene and mutations reported in Colorectal Adenocarcinoma TCGA PanCancer Atlas dataset, including 524 cases. (**B**) Distribution of EHMT2 genomic aberrations and mutations vs. mRNA expression (z-scores vs. diploid samples, RNA Seq V2 RSEM) in human colorectal tumors (*n* = 524 cases). (**C**) EHMT2 and APC mutation frequency from eight datasets. * A total of 3407 independent patients were queried due to patients overlap between some specific datasets. (**D**) Distribution of EHMT and/or APC-mutated cases across a dot chart of both genes’ expression levels (RSEM batch normalized from Illumina HiSeq_RNASeqV2) (*n* = 524 cases).
